# Annotations

**Published:** 1891-09-19

**Authors:** 


### Annotations.

**Doctors' Training in Lunacy.**

Any two medical practitioners can sign away a man's liberty by a simple certificate of insanity. It is not too much to say of a large proportion of the medical profession that they have no more knowledge or experience of insanity than have lawyers, clergymen, merchants, or any other class of persons who have received a good general education. This is a state of things highly discreditable to the legislature of this country, and more particularly to the General Medical Council. It will not be denied that it is a serious thing to certify a man a lunatic. All that anyone has to do is to suppose himself to be a little eccentric, a little out of health, and a subject for a lunacy certification. How would he like to be sat in judgment upon by some of the least reputable doctors of his own acquaintance? And yet that is what might easily happen to himself, and must constantly be happening to the eighty or ninety thousand poor persons who occupy our rate-supported lunatic asylums. It will hardly be believed by the laity that no medical student is compelled by law to have either training or examination in lunacy before acquiring his licence to practise and with it his right to give lunacy certificates. One of the greatest practical difficulties a medical student has to deal with is the opportunity of acquiring any knowledge of lunacy cases whatsoever. Yet, will it be credited, there are nearly ninety thousand lunatics maintained at the public expense, all of whose cases might render valuable service to medical education in this important specialty. The medical officers of the various asylums are anxious to have their institutions recognised as hospitals, and, wherever possible, to turn them into medical schools. This ought to be done with as little delay as possible. Every medical student should be required to attend for at least three months at a recognised lunatic asylum and to have taken out a course of not less than twenty systematic lectures. The asylums of the Local Government Board should be thrown open in all directions for post-graduate courses to duly qualified medical men, and certificates and liberal prizes should be offered to those practitioners who show special proficiency in the science and management of lunacy cases. An interesting paper on this subject was read before the members of the British Medical Association at Bournemouth, in July, by Dr. Francis Walmsley, of the Leavesden Asylum. The paper is published in the form of a pamphlet, and will well repay perusal. Dr. Walmsley is profoundly impressed by the widespread ignorance of lunacy cases which prevails in the medical profession.

**A Battle all but Won.**

Mr. James Matheson is engaged in the noble work of building a new hospital in the East-end of London, exactly where a hospital is needed. Mr. Matheson tells us that several deaconesses and doctors have been at work in rented premises in Bethnal Green for fifteen years past, "healing and soul-saving," as he expresses it. We confess our sympathy with both the healing and the soul-saving. Any form of Christianity cannot but be infinitely better than the dark and hopeless irreligion of much of the life of Eastern London. The new building is to be a mission hospital in connection with the Mildmay Park Conference Hall; and it is to be placed in Shoreditch. Towards the cost of erection thirteen thousand pounds have already been raised by Mr. Matheson and other friends of Evangelical religion. The Countess Tankerville has given and raised among her friends four thousand pounds. The late Mr. R. C. L. Bevan gave a thousand pounds, as have also Mr. John Deacon, Mr. Edward Rawlings, Mr. John Cunliffe, Mr. Gurney Sheppard, and Mr. Matheson himself. Miss Maynard and Miss L. Maynard, sisters-in-law of Mr. Matheson, have each given five hundred pounds, as also have the Rev. E. F. Wigram, of the Church Missionary Society, and Mr. T. A. Denny and his brother, Mr. E. M. Denny. The partners of Messrs. Brown, Shipley, and Co. have given six hundred and fifty pounds. Here, then, is a work of first-rate importance begun and carried almost to completion by a mere handful of well-known philanthropists, without blaze of trumpets or publicity of any kind. About four thousand pounds more are required to complete the hospital building. Mr. Matheson says he has exhausted the liberality of his own personal circle. He asks but four thousand pounds from outside sources for the completion of his building. For our part, we hope the public will give him not only four but fourteen thousand pounds more, and so provide him with a ten thousand pounds endowment for the carrying on of the work of the hospital when building operations shall have been completed. If anybody has a right to appeal to the public it is such men as Mr. Matheson and his coadjutors, who first put their hands so deeply into their own pockets before asking help outside. It will be noticed that many of those who have given so generously are well-known merchants. We make an earnest appeal to other generous representatives of the wealth of the City to complete the good work so nobly begun. Mr. Matheson may be found at 47, Phillimore Gardens, Kensington, W.

**Village Life.**

The *Daily News* has shown in a series of articles by a special commissioner how the dull season of the year may be utilised for purposes of the highest public importance. The anthropologist and the physiologist love to study man; the one having a great affection for fossil skulls and often misunderstood religions and customs; the other searching with painful minuteness for some small tag of hitherto undiscovered nerve fibre which shall hand down the name of the physiologist Smith to unborn generations. The anthropologist of the *Daily News* prefers to study man in our own beloved and honoured, though far from perfect, England. He has found out what a fine, honest, patient and steadfast race of men and women we have living, all unsuspected, at our own doors. He has brought to light the fact that the characteristics which have made the English statesman, soldier, merchant, and missionary famous all the world over, are the very characteristics which in the main distinguish the farm labourer and his wife in their village home. Fields of smiling corn and neatly built and beautifully thatched ricks on all hands attest the labourer's practical capacity. Cottages as clean and neat as hands can make them, with patches of bright flowers on odd bits of spare ground, show plainly his and his wife's love of colour and simple beauty. Children so clean, so neatly dressed, and so well behaved that one cannot but believe in miracles performed by mothers, are indisputable evidence of the innate and undying superiority of the English race. All these things have been brought to light by the village commissioner. It has also been shown how much of kindly feeling exists among landlords, clergy, and others of the more prosperous classes. In spite of all these things it is clear that villages and villagers are alike sinking into decay, and threatening in their fall to ruin the splendid agriculture of our country. This decay and threatened ruin are due to the one fact that the man who is born a labourer has, under our existing land system, practically no chance of ever becoming anything else but a labourer. The English labourer is an English man, and cannot, and will not do the best that is in him under conditions of hopeless slavery. He is not of the stuff of which slaves can be made. He is English. Give him a chance to show the real quality of the article. If land owners and legislators would but do this, they would soon put at least one fourth more rent into their own pockets, and they would restore for us a population of yeomen to give backbone to the country. Anthropology is like theology; it busies itself with the dead, and leaves the living to perish. A practical statesman who could furnish us with a workable scheme of land reform would be worth ten thousand anthropologists and theologians, whose work begins and ends with a learned published paper, or a sermon that is not learned and is not worth publication.

